# Identifying unreliable predictions in clinical risk models

**DOI:** 10.1038/s41746-019-0209-7

**Published:** 2020-01-23

**Authors:** Paul D. Myers, Kenney Ng, Kristen Severson, Uri Kartoun, Wangzhi Dai, Wei Huang, Frederick A. Anderson, Collin M. Stultz

**Affiliations:** 1 Department of Electrical Engineering and Computer Science and Research Laboratory for Electronics, Massachusetts Institute of Technology, Cambridge, MA USA; 2 Center for Computational Health, IBM Research, Cambridge, MA USA; 3 Center for Outcomes Research, University of Massachusetts Medical School, Worcester, MA USA; 4 Institute for Medical Engineering and Science, Massachusetts Institute of Technology, Cambridge, MA USA; 5 Division of Cardiology, Massachusetts General Hospital, Boston, MA USA

**Keywords:** Risk factors, Predictive markers, Prognostic markers

## Abstract

The ability to identify patients who are likely to have an adverse outcome is an essential component of good clinical care. Therefore, predictive risk stratification models play an important role in clinical decision making. Determining whether a given predictive model is suitable for clinical use usually involves evaluating the model’s performance on large patient datasets using standard statistical measures of success (e.g., accuracy, discriminatory ability). However, as these metrics correspond to averages over patients who have a range of different characteristics, it is difficult to discern whether an individual prediction on a given patient should be trusted using these measures alone. In this paper, we introduce a new method for identifying patient subgroups where a predictive model is expected to be poor, thereby highlighting when a given prediction is misleading and should not be trusted. The resulting “unreliability score” can be computed for any clinical risk model and is suitable in the setting of large class imbalance, a situation often encountered in healthcare settings. Using data from more than 40,000 patients in the Global Registry of Acute Coronary Events (GRACE), we demonstrate that patients with high unreliability scores form a subgroup in which the predictive model has both decreased accuracy and decreased discriminatory ability.

## Introduction

A necessary condition for the success of any predictive or classification model is that it achieves an accuracy that is superior to existing methods that are designed to accomplish the same task. In the healthcare sphere, however, accuracy alone does not ensure that a model will gain clinical acceptance. Unlike problems outside of the medical domain, poor performance for clinical models can have deleterious consequences for patients. The fact that a model may classify many patients correctly is certainly reassuring, but when the consequence of a misclassification is myocardial infarction, congestive heart failure, or death—typical outcomes of interest in the cardiovascular domain—it is important for clinicians to have some sense of when the predictive model will yield incorrect results. The accuracy, sensitivity, and specificity of a risk model provide information that can be used to estimate how often the model is likely to yield an incorrect result. However, as these metrics are calculated by evaluating the model’s performance on a range of patients, it is difficult to know how to leverage these data to identify specific patients or particular patient subgroups where model performance is likely to be reduced.

*Accuracy* reports the average performance on a dataset that contains a range of patient characteristics. High accuracy, however, does not ensure that the model will have adequate performance on distinct patient cohorts. For example, although the Framingham risk score—a widely used method to quantify the risk of developing atherosclerotic heart disease—has high accuracy overall, it may underestimate the risk of subclinical atherosclerosis in some women.^1^ Consequently, in this study, our goal is to identify a method that could identify, a priori, when a given patient belongs to a subgroup where the predictive model in question has reduced performance. We define predictions on patients who belong to these poorly performing subgroups as unreliable because they correspond to misleading statements about a given patient’s risk.

Previous methods that aspire to estimate prediction reliability can be grouped into two broad classes: model-dependent and model-independent approaches.^2^ Model-dependent methods generally report prediction confidence intervals that generally are calculated via least squares estimation or by estimating the uncertainty in learned model parameters.^3–6^ Some neural network models evaluate whether there are sufficient data in the training set to make a prediction for a test sample or whether the test sample is similar to a region of the training set where the model has poor performance.^7^ The drawback of these approaches is that they mandate the use of a particular type of classifier. Model-independent approaches, as the name implies, can be used with a variety of different predictive models, irrespective of the approach used to develop/train the model. Most model-independent approaches involve retraining the predictive model using an enhanced dataset that contains the original training set supplemented with new, unclassified data examples, where class labels for the unlabeled data are assigned based on the model’s predictions. The model’s performance before and after retraining are used to estimate the reliability of the predicted classes for the new data.^2,8,9^ New data that are similar to the original training data will therefore be more reliable in this framework, as adding data that are very similar to the training data will not yield a significantly different model. A disadvantage of these approaches is that, in practice, clinical datasets that are used to develop clinical risk scores are generally not available to users who would like to evaluate the reliability of a new prediction. Hence, retraining a model with new data (or directly assessing how different a new patient is from the training examples) is generally not possible, given the rightful concerns over guarding patient privacy. These approaches can therefore only be implemented by those who have access to the original dataset used to train the risk model in question. More importantly, even if such data were available, retraining complex models can be computationally expensive, thereby making this approach infeasible for the average user who has access to limited computational resources, or who requires some estimate of the reliability of a given patient’s prediction within a short time frame. A recently proposed model-independent approach, the “trust score”, does not require that the classifier be retrained.^10^ Nonetheless, to be computed it still requires access to the original training data, which may not be available to all health care providers who will use this metric. Furthermore, none of these approaches, neither model-dependent nor model-independent, have been evaluated in the setting of significant class imbalance. This is important because many clinical classification problems are associated with large class imbalance as the outcome of interest typically occurs in a small minority of patients.

Our goal was to develop a patient-specific metric that identifies when a given prediction is unreliable. In our view, a clinically useful prediction unreliability metric should: (1) first and foremost, identify patient subgroups that are associated with poor model performance; (2) be model independent; (3) not require retraining or access to the precise training dataset used to develop the original clinical risk model, thereby enabling the method to be implemented by practitioners who do not have access to the original data; and (4) be useful in the setting of significant class imbalance.

## Results

### Prediction unreliability is a function of class imbalance

**Fig. 1:**
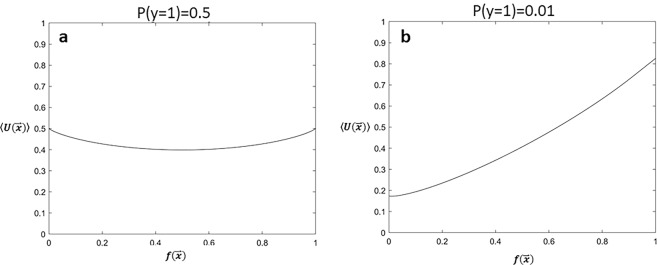
Unreliability scores are a function of class imbalance. **a** Average unreliability as a function of the classifier prediction, $f(\mathop{x}\limits^{\rightharpoonup} )$, when there is no class imbalance in the data, $P(y = 1) = 0.5$. **b** Average unreliability for different predictions, $f(\mathop{x}\limits^{\rightharpoonup} )$, in the setting of large class imbalance, $P(y = 1) = 0.01$. Calculated expected values assume a uniform distribution for $\beta _{\mathop{x}\limits^{\rightharpoonup} }$.

The unreliability metric, $U( {\mathop{x}\limits^{\rightharpoonup} })$, is a function of the risk model, $f(\mathop{x}\limits^{\rightharpoonup} )$, the prevalence of the outcome of interest in the overall population, *P*(*y* = 1), and the relative likelihood ratio of the positive and negative classes arising from the generative models, $\beta _{\mathop{x}\limits^{\rightharpoonup} }$ (see Methods and Supplementary Methods). To understand how each of these quantities affects the unreliability estimate, we computed $U( {\mathop{x}\limits^{\rightharpoonup} })$ for a range of input parameters and then calculated the average value of $U( {\mathop{x}\limits^{\rightharpoonup} })$ as a function of the risk model prediction. We consider two limiting cases: (i) when there is no class imbalance in the training data ($P(y = 1) = 0.5$, Fig. 1a), and (ii) when the positive class (*y* = 1) is in the minority ($P(y = 1) = 0.01$, Fig. 1b). When there is no class imbalance on average, values of the clinical risk score that are close to 0 or 1 are slightly more unreliable than values close to 0.5, which is equal to the prevalence of the outcome in the population (Fig. 1a). By contrast, on average, when there is significant class imbalance, unreliable predictions are more likely to occur in patients who are predicted to be at high risk (Fig. 1b). In other words, when there is a relatively small number of patients who belong to the positive class, and consequently few positive examples for the clinical model to learn from, positive predictions are, on average, more likely to have high values of $U( {\mathop{x}\limits^{\rightharpoonup} })$.

### Performance of the GRACE score in an unreliable subgroup

We hypothesize that patients with high unreliability scores form a subgroup in which it is particularly difficult to correctly assess their risk, and that the clinical risk model under scrutiny would therefore have decreased accuracy in cohorts enriched with patients who have unreliable predictions.

To test whether unreliable predictions result in decreased accuracy, we computed $U( {\mathop{x}\limits^{\rightharpoonup} })$ for an established, and widely used, clinical risk score. Using the Global Registry of Acute Coronary Events (GRACE) risk score, which quantifies the risk of death 6 months after presenting with an acute coronary syndrome,^11^ our goal was to compute $U( {\mathop{x}\limits^{\rightharpoonup} })$ for patients in the GRACE registry, identify the most unreliable predictions in this cohort, and evaluate the performance of the GRACE score on this “unreliable” subgroup.

**Fig. 2:**
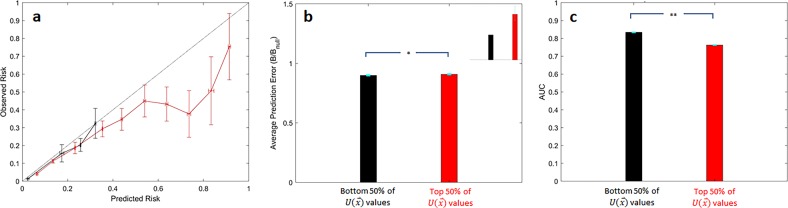
Prediction scores in the upper 50th percentile of unreliability (top 50% of $U(\mathop{x}\limits^{\rightharpoonup} )$ values) have worse performance. **a** Calibration curves, **b** Normalized Brier scores (inset shows expanded region corresponding to $0.89 \le {\mathrm {Brier}}/{\mathrm {Brier}}_{{\mathrm {null}}} \le 0.912$), and **c** Average AUCs for predicting the outcome of all-cause death within 6 months of presentation for predictions in the upper 50th percentile (red) and those within the lower 50th percentile (black). *p < 0.05; **p << 0.0001. Error bars are from 100 bootstrap splits and show one standard deviation (**a**) or one standard error of the mean (**b** and **c**).

We computed $U( {\mathop{x}\limits^{\rightharpoonup} })$ for all patients in the GRACE test set and evaluated the accuracy of predictions that have high unreliability relative to those who have a lower unreliability score. First, we note that, consistent with the data shown in Fig. 1b, as there is considerable class imbalance in the GRACE dataset, patients who are predicted to be high risk are more likely, on average, to have high unreliability scores (Fig. 2a and Supplementary Fig. 1). Calibration curves demonstrate that patients within the upper 50% of unreliability values tend to overestimate a patient’s risk of death (Fig. 2a, red curve), while predictions within the lower 50% of unreliability values are well calibrated (Fig. 2a, black curve).

To more quantitatively assess the accuracy of predictions arising from the GRACE score in unreliable patient subgroups, we rely on the Brier score—a popular metric that quantifies the expected classification error.^12,13^ Formally, the Brier score is the mean square error between the prediction and the true class label; i.e., lower Brier scores indicate more accurate predictions. However, the Brier score itself is dependent on the prevalence of the outcome of interest, and consequently can be difficult to interpret when assessing the performance of a classifier on subgroups that have different expected outcome rates; i.e., the Brier score tends to be lower when the incidence of the outcome is low.^14^ Therefore, we normalize the Brier score by a Brier_null_ model, which corresponds to the scenario where every patient is predicted to have a risk equal to the prevalence of the outcome in that respective subgroup. Similar approaches have been used to assess the performance of prediction models in the setting of class imbalance.^14,15^ Predictions corresponding to the top 50% of $U( {\mathop{x}\limits^{\rightharpoonup} })$ values have a modest, yet statistically significant, reduced accuracy, as measured via a normalized Brier score, relative to predictions that fall within the lower 50% of $U( {\mathop{x}\limits^{\rightharpoonup} })$ values (Fig. 2b, and inset).

As high unreliability scores are associated with decreased predictive accuracy, we hypothesize that the clinical risk model under scrutiny would also have poor discriminatory ability in cohorts enriched with patients with unreliable predictions. Hence, we computed the AUC (or C-statistic) for patients to assess the discriminatory ability of both the GRACE and Stroke risk scores patients who have unreliable predictions. The GRACE score similarly has relatively poor discriminatory ability in predictions that fall within the top 50% of $U( {\mathop{x}\limits^{\rightharpoonup} })$ values (Fig. 2c).

### The most unreliable GRACE predictions have significantly reduced performance

**Fig. 3:**
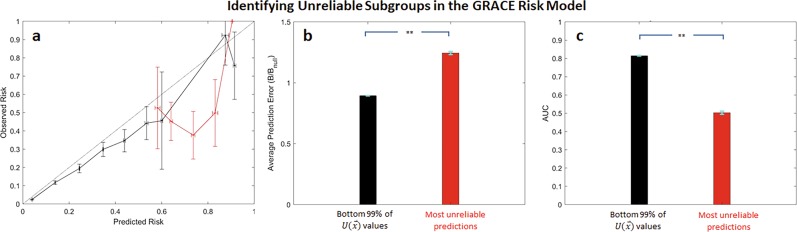
The most unreliable predictions (predictions within the top 1% of $U(\mathop{x}\limits^{\rightharpoonup} )$ values) in the GRACE risk model are associated worse performance. **a** Calibration curves, **b** normalized Brier scores, and (c) AUCs for the most unreliable predictions (red) and those that fall within the bottom 99% of $U(\mathop{x}\limits^{\rightharpoonup} )$ values (black). **p << 0.0001. Error bars are from 100 bootstrap splits and show one standard deviation (**a**) or one standard error of the mean (**b** and **c**). Error bars are truncated at 1.

In light of these data, we evaluated the relative performance of predictions that have very high unreliability values. Calibration curves for predictions that fall within the top 1% of $U( {\mathop{x}\limits^{\rightharpoonup} })$ values (henceforth referred to as the “most unreliable predictions”) similarly underestimate the actual risk of death (Fig. 3a, red curve). It is important to note that while $U( {\mathop{x}\limits^{\rightharpoonup} })$, on average, tends to assign high values to high-risk predictions, not all high-risk predictions are assigned to the most unreliable subgroup (Fig. 3a). The method preferentially identifies those predictions that most differ from the observed risk.

Similarly, the prediction error, measured via a normalized Brier score, for the subgroup consisting of the most unreliable predictions is significantly higher than the error associated with all other patients in the dataset (Fig. 3b). The AUC for the most unreliable subgroup is also significantly reduced relative to the AUC of the remainder of the test data set, suggesting that predictions for the most unreliable subgroup have significantly reduced discriminatory ability relative to the remainder of the dataset (Fig. 3c).

To assess whether these findings are unique to our unreliability metric, or whether they generalize to other metrics that strive to quantify the reliability in a given risk prediction, we computed an alternate metric that purports to quantify when a given classifier’s result should be trusted. The *trust score* measures the agreement between the classifier and a nearest-neighbor classifier on a testing example. At a high level, the score measures the distance between a given test set example and training examples in each class; i.e., the set consisting of training examples that have the outcome of interest and the set of training examples that do not. For a binary classification problem, the trust score is the ratio between the distance to the alternate class and the distance between the predicted class. Unlike our unreliability metric, which associates high values of $U( {\mathop{x}\limits^{\rightharpoonup} })$ with unreliable predictions, low trust scores denote predictions that are untrustworthy as they are more similar to training examples in the class that is different from the one that the model predicts.^10^

**Fig. 4:**
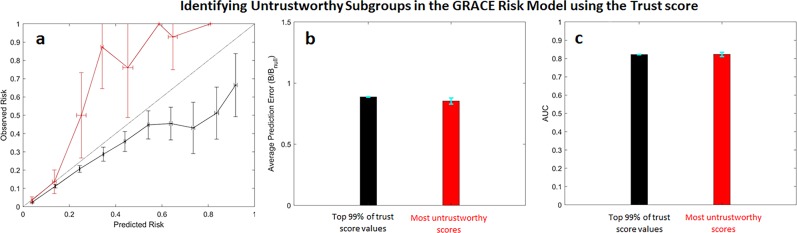
Untrustworthy predictions in the GRACE risk model using the trust score. **a** Calibration curves, **b** normalized Brier scores, and **c** AUCs for the most untrustworthy predictions (red) and those that fall within the bottom 99% trust score values (black). Error bars are from 100 bootstrap splits and show one standard deviation (**a**) or one standard error of the mean (**b** and **c**). Error bars are truncated at 1.

Calibration curves for the most untrustworthy predictions (i.e., predictions within the lowest 1% of trust values) demonstrate that the method identifies inaccurate predictions. Predictions within the top 1% of trust score values underestimate the actual patient risk (Fig. 4a, red curve). However, predictions in the remainder of the dataset tend to overestimate patient risk (Fig. 4a, black curve). Moreover, normalized Brier scores suggest that the most untrustworthy predictions have errors that are similar to that of predictions in the remainder of the dataset (Fig. 4b). Calculated AUCs of both subgroups suggest that the discriminatory ability of the classifier is similar in both the most untrustworthy subgroup and in the subgroup containing all other remaining patients.

### Unreliable predictions in a stroke risk model

**Fig. 5:**
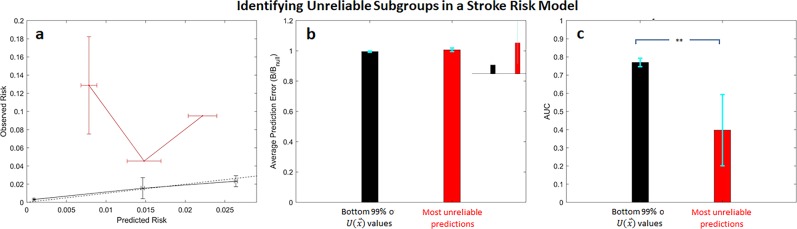
Unreliable predictions in the Stroke risk model. **a** Calibration curves, **b** normalized Brier scores (inset shows expanded region corresponding to $0.95 \le {\mathrm {Brier}}/{\mathrm {Brier}}_{{\mathrm {null}}} \le 1.02$), and **c** AUCs for the most untrustworthy predictions (red) and those that fall within the bottom 99% $U(\mathop{x}\limits^{\rightharpoonup} )$ values (black). **p « 0.0001. Error bars are from 100 bootstrap splits and show one standard deviation (**a**) or one standard error of the mean (**b** and **c**).

To determine whether our findings generalize to other outcomes and risk scores, we developed a model to predict the risk of in-hospital stroke in patients presenting with an acute coronary syndrome. Unreliability scores were again computed for all patients using Eq. (2). The most unreliable patients form a subgroup whose risk is underestimated by the model (Fig. 5a), while the model is well calibrated for all other patients in the dataset. The average error for patients in the most unreliable subgroup is larger than the average error for other patients in the dataset, as expected, although this difference is not statistically significant (Fig. 5b and inset). The discriminatory ability of the classifier is reduced in the most unreliable cohort, relative to the classifier’s discriminatory ability in the remainder of the data (Fig. 5c).

**Fig. 6:**
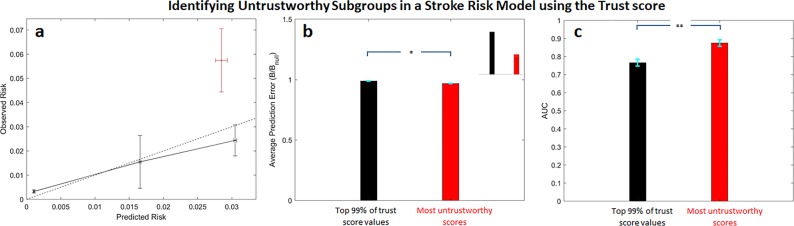
Untrustworthy predictions in the Stroke risk model using the trust score. **a** Calibration curves, **b** normalized Brier scores (inset shows expanded region corresponding to $0.95 \le {\mathrm {Brier}}/{\mathrm {Brier}}_{{\mathrm {null}}} \le 1.0$), and **c** AUCs for the most untrustworthy predictions (red) and those that fall within the bottom 99% $U(\mathop{x}\limits^{\rightharpoonup} )$ values (black). *p < 0.05, **p « 0.0001. Error bars are from 100 bootstrap splits and show one standard deviation (**a**) or one standard error of the mean (**b** and **c**).

We again computed trust score values for the Stroke risk model predictions and evaluated the performance of the subgroup consisting of the most untrustworthy predictions. Similar to what was observed with the trust calculations on the GRACE risk model, predictions with the highest trust score underestimate the overall risk of stroke (Fig. 6a). However, the average prediction error associated with this untrustworthy subgroup is lower than that of the other patients in the dataset, and the corresponding difference is statistically significant (Fig. 6b and inset). Moreover, the discriminatory ability of the stroke risk score is actually higher in the untrustworthy subgroup (Fig. 6c).

## Discussion

While a great deal of effort has been devoted in recent years to constructing clinical classifiers that have improved discriminatory ability, relatively little work had been devoted to developing methods that help health care providers determine when a given prediction is likely to be useful. While the overall accuracy and the AUC are important measures that gauge a model’s performance, these metrics are obtained by averaging over a range of patients in a pre-specified dataset. Identifying subgroups where model performance is reduced is inherently challenging using these standard statistical metrics of success alone. Nevertheless, understanding when the output from a prediction model can be trusted is an important clinical problem in itself. Demonstrably good overall performance on a specified dataset helps to ensure that the risk model will, on average, perform well. This does not, however, help the clinical provider identify patient subgroups where the predictive model is expected to perform poorly.

In this work, we developed a method that identifies cohorts that are associated with poor model performance. Our approach identifies unreliable predictions by comparing a given prediction for a patient, using the predictive model of interest, to another prediction arising from an alternate risk model that was derived from the same training data. If the two predictions disagree, then we say that the training data are insufficient to yield a robust prediction for that patient. Hence, the method infers the adequacy of the training set for making predictions about a given patient.

Our method is model independent, in that it can be used on any clinical risk model, irrespective of the method used to derive the model. In addition, the method does not require access to the precise training data to assess model reliability—only summary statistics from the training data are needed. Moreover, in the setting of significant class imbalance, subgroups enriched with unreliable predictions, on average, form a heterogeneous cohort of patients (see Supplementary Table 1 and Supplementary Figs 1 and 2), which is more likely to contain patients that the risk model *believes* to be high risk (Fig. 1b, and Supplementary Fig. 1). This is appropriate because a dearth of examples in the minority class means that the risk model has relatively few examples to “learn” the relationship between prognostic features and patient risk.

High unreliability scores identify patient subgroups where: (1) the predicted outcome rates differ from the corresponding observed outcome rates in the data, and (2) the predictive model has decreased accuracy and poor discriminatory ability relative to predictions that have lower unreliability. To determine whether our findings are unique to our method, or whether they generalize to other methods that strive to predict prediction reliability, we compared our method to a recently described approach—the trust score—for identifying trustworthy predictions.^10^ The most untrustworthy predictions, identified using the trust score, for the GRACE and Stroke risk models correspond to patients whose risk is underestimated by the relevant risk model (Figs 4a and 6a). However, for the GRACE score, predictions that are more trustworthy tend to overestimate patient risk (Fig. 4a), and the overall accuracy and discriminatory ability of the trust score for the least trustworthy predictions is similar to, or better than, more trustworthy predictions (Figs 4c, 6c). By contrast, unreliable predictions, identified using our unreliability score, form subgroups where the predictive model has reduced discriminatory ability in all of the patient subgroups that we studied.

In principle, a risk model will have poor discriminatory performance on a given dataset when: (1) the model itself is incorrect (e.g., the model parameters are wrong) or (2) the model is applied to a cohort that has a distribution of patient characteristics that is very different from the distribution of data in the original training/development dataset.^16^ More precisely, a given training/development set is drawn from some underlying distribution of clinical interest (e.g., patients who present an acute coronary syndrome). While an accurate model will perform well, on average, when applied to a large number of patients drawn from this distribution, it is not guaranteed to perform well when applied to a cohort that has a very different distribution of characteristics; i.e., poor performance on a given subgroup does not necessarily mean that the model is “wrong” for the original patient distribution. Nevertheless, predictions arising from risk models applied to cohorts drawn from a different distribution of patient characteristics should likewise not be trusted. In future applications, especially with risk models that have not been extensively validated on different external datasets, additional studies may be required to clarify whether model correctness vs. changes in the underlying patient distribution is most responsible for the reduced performance.^16,17^

A limitation of our method is that only large values of $U( {\mathop{x}\limits^{\rightharpoonup} })$ are informative; i.e., large values of $U( {\mathop{x}\limits^{\rightharpoonup} })$ are associated with decreased accuracy and discriminatory ability. By contrast, small values of $U( {\mathop{x}\limits^{\rightharpoonup} })$ do not necessarily mean that $f(\mathop{x}\limits^{\rightharpoonup} )$ accurately predicts the risk of an adverse outcome for a patient with prognostic features, $\mathop{x}\limits^{\rightharpoonup}$. In particular, $U( {\mathop{x}\limits^{\rightharpoonup} }) = 0$ when the risk model agrees with the prediction from the prediction arising from the alternate model; i.e., $f( {\mathop{x}\limits^{\rightharpoonup} }) = P^G\left( {y = 1|\hat y} \right)$. If the set of prognostic features for a given patient, $\mathop{x}\limits^{\rightharpoonup}$, does a poor job of distinguishing between the positive and negative classes, then $f(\mathop{x}\limits^{\rightharpoonup} )$will be a poor classifier for this patient, even when $U( {\mathop{x}\limits^{\rightharpoonup} }) = 0$. Large values of $U( {\mathop{x}\limits^{\rightharpoonup} })$ are of interest because they suggested that the risk model in question is likely to be less useful than one would gauge from an analysis of the model’s performance on a large dataset. Health care providers should therefore view predictions with unreliability scores above the 1 percentile ($U( {\mathop{x}\limits^{\rightharpoonup} }) > 0.11$ for GRACE) with care and obtain additional data, possibly arising from other risk metrics, to arrive at a more accurate assessment of that patient’s risk. Another limitation of our study is that the cohorts used to validate the method were derived from a common data resource, GRACE. While GRACE is large registry that contains patients who have a wide range of clinical characteristics, it, like all other registries, has its own biases. For example, in both the ACS and Stroke cohorts, only ~30% of the patients are women (Table 1). Application to additional datasets will help to clarify the types of outcomes and data that are most relevant to our method. Nevertheless, our results highlight the promise of our approach for evaluating the reliability of predictions arising from models designed to quantify the risk of adverse cardiovascular outcomes.

**Table 1:** Population characteristics in the subset of the GRACE dataset used for all analyses.

	Dataset for GRACE risk model	Dataset for in-hospital Stroke risk model
Population size	43,063	16,618
Mortalities	3078 (7.15%)	85 (0.511%)
Demographics		
Age in years (IDR)	66.1 (43.6–86.1)	65.1 (50.3–87.7)
Female	32.6%	31.8%
Height in cm (IDR)	170 (152–183)	169 (154–180)
Admission weight in kg (IDR)	77.0 (53.0–109.4)	79.2 (50.0–99.5)
*Medical histor*y		
Including cardiac risk factors (%)		
Angina	51.9	42.3
Congestive heart failure	10.5	10.1
Coronary artery bypass graft	12.6	12.2
Diabetes	25.1	25.0
Hyperlipidemia	48.3	51.4
Hypertension	62.1	62.6
Myocardial Infarction	30.3	28.0
Percutaneous coronary intervention	17.7	19.6
Peripheral artery disease	9.7	9.3
Renal insufficiency	7.8	7.5
Smoking	57.7	58.1
TIA/Stroke	8.3	8.1

*IDR* interdecile range

In this work, we used a multivariate normal (MVN) distribution as our generative model to calculate the relative likelihood, $\beta _{\mathop{x}\limits^{\rightharpoonup} }$. However, our unreliability approach is agnostic to the specific model used to calculate the relative likelihood and can leverage any generative model. Assumptions made about the distribution of the data are isolated to the choice of generative model and are not fundamental to the method itself. More sophisticated models for the positive and negative class may be required when the training data are not well described by Gaussian probability density functions.

We approached the problem of identifying unreliable predictions by first identifying criteria that would maximize the clinical utility of a potential unreliability metric. In our view, a clinically useful unreliability metric should identify particular patient subgroups where the accuracy, discriminatory ability, and calibration of the risk model are compromised. Risk models, for example, that are unable to discriminate between high-risk and low-risk patients within a particular subgroup, should not be used for patients who belong to that subgroup, as such results are potentially harmful to that patient. This model-independent approach identifies patient subgroups where the model has demonstrably reduced performance, and that directly relates model unreliability to clinical metrics of performance using real patient data. Such approaches will become increasingly important as clinical risk metrics rely on more complex machine learning methods that provide little clinical insight into how they arrive at a particular result.

## Methods

### A method for estimating unreliable predictions

Given a clinical risk stratification model that estimates the probability of an adverse outcome for a patient with a set of prognostic features, our strategy for identifying unreliable predictions is to construct a separate risk metric using summary statistics from the same training data that were used to develop the risk model under consideration. When the two estimates disagree on a specific patient, we say that the training data are unable to provide a reliable prediction for this patient. To make this explicit, let:

- $\mathop{x}\limits^{\rightharpoonup}$ denote a random variable corresponding to a set of prognostic characteristics (the feature vector);
- $y{\it{\epsilon }}$ 0,1 denote a random variable designating the true patient outcome (also known as the class label); i.e., we consider a binary classification problem. For example, *y* = 1 if a patient dies within some specified time after initial diagnosis (the positive class) and *y* = 0 otherwise (the negative class);
- $f(\mathop{x}\limits^{\rightharpoonup} )$ denote the clinical risk model that takes a feature vector as input and outputs a risk score that can be used to estimate the probability of the true class label. As clinical risk models generally report the probability of an adverse event, or some score that can be translated into a probability, via a nomogram, we consider the case where $0 \le f(\mathop{x}\limits^{\rightharpoonup} ) \le 1$.

The separate, alternate, risk metric, $P^G(y = 1|\mathop{x}\limits^{\rightharpoonup} )$, is calculated using the same data that were used to calculate $f(\mathop{x}\limits^{\rightharpoonup} )$. As we are interested in developing a method that does not require training a new model using the training data, our goal is to derive an expression for $P^G(y = 1|\mathop{x}\limits^{\rightharpoonup} )$ that is straightforward to compute based only on summary statistics of the training data. In our formalism, $P^G(y = 1|\mathop{x}\limits^{\rightharpoonup} )$ is calculated using appropriately trained generative models where one model generates feature vectors consistent with patients in the positive class, $\mathop{x}\limits^{\rightharpoonup} |y = 1$, and the other generates feature vectors consistent with patients in the negative class, $\mathop{x}\limits^{\rightharpoonup} |y = 0$ (hence the superscript *G*). With these conventions, the corresponding unreliability metric can be represented as:

1 $$\upsilon (\mathop{x}\limits^{\rightharpoonup} ){\mathrm{ }} = \left| {P^G\left( {y = 1|\mathop{x}\limits^{\rightharpoonup} } \right) - \hat y} \right|,$$

where $\hat y = f(\mathop{x}\limits^{\rightharpoonup} )$. It follows that $0 \le \upsilon (\mathop{x}\limits^{\rightharpoonup} ) \le 1$, where the higher the value of $\upsilon (\mathop{x}\limits^{\rightharpoonup} )$ the more unreliable the model prediction. In practice, we work with an alternate form of $\upsilon (\mathop{x}\limits^{\rightharpoonup} )$, which is similar to that presented in equation,^18^ but that is easier to calculate:

2 $$U( {\mathop{x}\limits^{\rightharpoonup} }) = \left| {P^G\left( {y = 1|\hat y} \right) - \hat y} \right|,$$

where $P^G\left( {y = 1|\hat y} \right)$ is the probability that a patient actually belongs to the *y* = 1 class, given that the clinical risk model, $f(\mathop{x}\limits^{\rightharpoonup} )$, assigns a score of $\hat y$ to a patient with prognostic features, $\mathop{x}\limits^{\rightharpoonup}$ (see Supplementary Methods). It is straightforward to show that $U(\mathop{x}\limits^{\rightharpoonup} ) \ne 0$ implies $P^G( {y = 1{\mathrm{|}}\mathop{x}\limits^{\rightharpoonup} }) \ne f(\mathop{x}\limits^{\rightharpoonup} )$ (see Supplementary Methods).

### Clinical risk models and data

All experiments were done using the GRACE dataset.^11,19,20^ GRACE was designed to reflect an unbiased and generalizable sample of ACS patients hospitalized from 1999 to 2007 in 94 hospitals in 14 countries. All methods were carried out in accordance with relevant guidelines and regulations at each participating site, and only patients ≥18 years of age were eligible to be enrolled in the database.^21^ The GRACE protocol was approved by the UMass Medical School institutional review board and participating hospitals, where required, also received approval from their local ethics or institutional review boards. Signed, informed consent for follow-up contact was obtained from the patients at enrollment. For those sites using active surveillance for case identification, verbal or written consent was obtained from patients to review information contained in their medical charts. Details of the GRACE design, recruitment, and data collection are described elsewhere.^11,21–24^

We considered two risk models in this study: The GRACE score and a Ridge Logistic Regression (Stroke risk) model that we trained on the GRACE dataset to predict in-hospital stroke (the Stroke risk model or SRM).

We identified a subset of patients in the GRACE dataset consisting of patients who have values for all features used in the GRACE risk model. This dataset consists of a set that was used to develop the GRACE risk score (i.e., the original GRACE training/development set) and an additional set of patients that we use to evaluate the GRACE metric and our unreliability score (i.e., the GRACE test set). The all-cause mortality rate within the GRACE dataset was 7.15% (Table 1). To use the model, we converted the raw GRACE score to a probability using the published nomogram. We found that the GRACE score to be well calibrated over the entire dataset as assessed using a Hosmer–Lemeshow test.

In addition to all-cause mortality, we constructed a separate dataset to predict in-hospital stroke using a feature set distinct from the GRACE set of predictive features. Features in this separate dataset included all available information (198 features) during the first 24 h after hospital admission and the patients with no missing values were selected. These features collectively include laboratory data, patient demographic information, as well medications administered during the first hospital day.

### Calculating ${\boldsymbol{U}}( {\mathop{{\boldsymbol{x}}}\limits^{\rightharpoonup}})$

To compute $U( {\mathop{x}\limits^{\rightharpoonup} })$ for a given patient, two quantities are required: $f(\mathop{x}\limits^{\rightharpoonup} )$ and $P^G\left( {y = 1|\hat y} \right)$. The classifier, $f(\mathop{x}\limits^{\rightharpoonup} )$, corresponds to either the GRACE score, after converting it to a probability using a published nomogram,^23^ or the output of the Stroke risk model. $P^G\left( {y = 1|\hat y} \right)$ is calculated from Bayes’ rule using the probabilities arising from a generative model (i.e., $P^G\left( {\hat y|y = 1} \right)$ and $P^G\left( {\hat y|y = 0} \right)$), and *P*(*y* = 1), which corresponds to the fraction of patients in the dataset who have the outcome of interest (see Supplementary Methods). In practice, we use the ratio of the likelihoods from the generative model, $\beta _{\mathop{x}\limits^{\rightharpoonup} } \equiv {\textstyle{{P\left( {\hat y|y = 1} \right)} \over {P\left( {\hat y|y = 0} \right)}}}$, a quantity we call the relative likelihood (see Supplementary Methods). For these calculations, we used a MVN distribution probability density function (PDF) to calculate generative likelihoods as this provides an efficient, and widely used, mechanism for likelihood estimation.

To estimate the relative likelihood, $\beta _{\mathop{x}\limits^{\rightharpoonup} }$, we separated the training data into positive and negative patients, and then fit separate MVNs to the two patient populations; the mean and covariance were estimated using the sample mean and sample covariance, respectively. When calculating $P^G(\hat y|y = 1)$ and $P^G(\hat y|y = 0)$ for the GRACE score, we calculated mean and covariance matrices using a dataset consisting of those that were originally used to develop the GRACE score (13,777 patients, 7.5% mortality rate). To evaluate our unreliability score, we did bootstrapping by randomly sampling 20% of the remaining patients (29,286 patients, 7.0% mortality rate) to form the test sets. One-hundred bootstraps were performed to obtain statistical measures of uncertainty. A MVN was also used to model the positive and negative patient distributions for the Stroke risk model. However, given the small event rate (~0.5%) in this dataset, we used three bootstrapped test datasets.

For both the GRACE score and the Stroke risk model, $P^G(\hat y|y = 1)$ and $P^G(\hat y|y = 0)$ were numerically calculated by randomly sampling 10^6^ feature vectors, $\vec x$, from each distribution, passing each vector through either the GRACE or Stroke risk models to get a corresponding $\hat y$ value for each feature vector, and finally by constructing a histogram to estimate the PDFs corresponding to $P^G(\hat y|y = 1)$ and $P^G(\hat y|y = 0)$. Histograms were constructed using bins of width 0.001; continuous features are normalized to lie between 0 and 1 and binary features were treated as arising from an underlying continuous distribution with thresholding. Although the GRACE score does include three binary features and one categorical feature (which is equivalent to including additional binary features using a one-hot encoding), a MVN approximation adequately represents the feature space. Indeed, using a MVN to model all of the GRACE score features (both binary and continuous features) yields an AUC for $P^G\left( {y = 1|\hat y} \right)$ of 0.8123, which was similar to the AUC of the GRACE risk model (0.8124).

This process was repeated with another set of 10^6^ samples, which were combined with the previous 10^6^ samples. If the combined histogram and previous histogram were found to be different according to a two-sample Kolmogorov–Smirnov (K–S) test, the process was repeated until there was no change in the histogram according to the K–S test; the PDFs were then said to have converged.

### Trust score calculations

We evaluated the performance of an alternate metric for quantifying the trustworthiness of a given prediction.^10^ The method involves: (1) pre-processing the training set to first identify training examples in both classes; i.e., patients that have the outcome of interest and the distance from training examples that do not; (2) removing outliers from both classes; and (3) measuring the distance of a given test set example from both classes. The resulting *trust score* is a ratio of both distances.

### Statistical analysis

For both the GRACE risk and Stroke risk models, bootstrapping (sampling with replacement) was performed to obtain statistical measures of performance. For both risk models, a bootstrapped set was 20% the size of the test set and was stratified for the outcome of interest; i.e., all-cause mortality for the GRACE risk model and in-hospital stroke for the Stroke risk model. All statistical testes were two-sided, paired-sample *t*-tests among the 100 bootstrapped datasets for calculations involving the GRACE score, and among three bootstrapped datasets (due to the much smaller prevalence of the outcome of interest) for calculations involving the Stroke risk model. The average model error (also known as the Brier score) is calculated using the formula $B = \frac{1}{N}\mathop {\sum }\limits_{i = 1}^N (y_i - f(\mathop{x}\limits^{\rightharpoonup} _i))^2$, where *y*_*i*_ is the true class label. Brier scores are scaled by the average error of a model that predicts every patient to have a risk equal to the prevalence of the outcome in the population of interest. The normalized Brier score is equal to *B*/*B*_null_ where $B_{{\mathrm {null}}} = \frac{1}{N}\mathop {\sum }\limits_{i = 1}^N (y_i - \bar y)^2$ and $\bar y$ is the expected outcome prevalence in the relevant patient subgroup. Unlike the Brier score, which is bounded by 0 and 1, normalized Brier scores can be greater than 1 (see Supplementary Methods). Normalized Brier scores and AUCs for different subgroups are shown as mean ± the standard error of the mean.

Calibration curves for the GRACE data were obtained by first binning the predictions into 10 bins centered at 0.05,0.15,0.25…,0.95. The fraction of patients who died was then computed for each bin. This process was repeated for each of the 100 bootstraps for the GRACE risk model. For a given bootstrap, the resulting calibration data correspond to the average value of the prediction within each bin (x-axis) vs the fraction of patients, who were assigned to that bin, who died (y-axis). Data for all bootstraps are shown as the mean ± standard deviation. For the Stroke model, a similar procedure was applied, except that the only difference was that three equally spaced bins centered at 0.005, 0.015, and 0.025 (the maximum value of the Stroke risk model output is ~0.03) were used for each bootstrap because of the dearth of positive examples in the dataset, and only three bootstraps were performed.

### Reporting summary

Further information on research design is available in the Nature Research Reporting Summary linked to this article.

## Supplementary information

Supplementary Information

Reporting Summary Checklist

## Data Availability

The GRACE dataset is available from Center for Outcomes Research, University of Massachusetts Medical School, but restrictions apply to the availability of these data. Data are, however, available upon reasonable request and with permission from the Center for Outcomes Research.
