# Supplementary material for: Identifying unreliable predictions in clinical risk models
Source: NPJ Digit Med. 2020 Jan 23;3:8. doi: 10.1038/s41746-019-0209-7 (PMC6978376; doi:10.1038/s41746-019-0209-7)
Supplement: Supplementary file 1 — Supplementary Information [file 41746_2019_209_MOESM1_ESM.pdf]

Supplementary Information for

Identifying Unreliable Predictions in Clinical Risk Models

Paul D. Myers, Kenney Ng, Kristen Severson, Uri Kartoun, Wangzhi Dai, Wei Huang, Frederick A. Anderson & Collin M. Stultz

SUPPLEMENTARY METHODS

I. Theory: A simplified Formula for Calculating $U(\vec{x})$

Let:

- $\vec{x}$ denote a random variable corresponding to a set of prognostic characteristics (the feature vector);
- $y \in \{0,1\}$ denote a random variable designating the true class label; i.e., we consider a binary classification problem. For example, $y = 1$ if a patient dies within some specified time after initial diagnosis (the positive class) and $y = 0$ otherwise (the negative class);
- $f(\vec{x})$ be the clinical risk model that takes a feature vector as input and outputs a risk score that can be used to estimate the probability of the true class label.

As clinical risk models generally report the probability of an adverse event, or some score that can be translated into a probability, we consider the case where $f(\vec{x})$ is a probability; i.e., $f(\vec{x}) = P^E(y = 1|\vec{x})$, where $P^E(y = 1|\vec{x})$ is the probability that $y = 1$ for a patient with feature vector $\vec{x}$. The superscript E designates that this probability is estimated from a training dataset. Our alternate metric, $P^G(y = 1|\vec{x})$, uses the same data that was used to calculate $f(\vec{x})$ and is calculated using generative models for both the positive and negative classes (hence the superscript G). When $f(\vec{x})$ and $P^G(y = 1|\vec{x})$ disagree, we say that the training data are insufficient to provide a robust, trustworthy prediction.

As we are interested in developing a method that does not require training a new model using the training dataset, our goal is to derive an expression for $P^G(y = 1|\vec{x})$ that is straightforward to compute. Using Bayes' rule, we can express $P^G(y = 1|\vec{x})$ as follows:

$$P^G(y=1|\vec{x}) = \frac{P^G(\vec{x}|y=1)P(y=1)}{P^G(\vec{x}|y=1)P(y=1) + P^G(\vec{x}|y=0)(1-P(y=1))} \quad (1)$$

where $P(y = 1)$ is the prevalence of the adverse outcome in the overall population and is estimated using the fraction of patients who belong to the positive class in the training dataset. Calculating $P^G(y = 1|\vec{x})$ requires us to estimate the likelihood of observing a given feature vector in the positive class, $P^G(\vec{x}|y = 1)$, and the negative class, $P^G(\vec{x}|y = 0)$. These likelihoods can be estimated given appropriately trained generative models where one model generates feature vectors consistent with patients in the positive class, $\vec{x}|y = 1$, and the other generates feature vectors consistent with patients in the negative class, $\vec{x}|y = 0$. In practice, $P^G(\vec{x}|y = 0)$ is agnostic to the type of generative model used. In this initial work we use a multivariate normal (MVN) probability density function (pdf) for our generative model. As outlined in the main text, this model forms a good model for the feature space as using a MVN to model all of the GRACE score features (both binary and continuous features) yields an AUC for $P^G(y = 1|\hat{y})$ is 0.8123, which is similar to the AUC of the GRACE risk model (0.8124).

In practice, using equations (1) requires us to calculate definite integrals of multidimensional Gaussian pdfs, as the probabilities correspond to integrals over the pdfs. However, calculating definite integrals of MVN densities can be challenging given their computational complexity[1]. For example, if $\dim(\vec{x}) = N$ then numerical estimates of the pdf would require sampling to ensure convergence for 10^N values, where each prognostic feature is partitioned into 10 bins. To simplify the calculations, we therefore rewrite the probability estimate so that it only requires the numerical calculation of a univariate probability density:

$$P^G(y=1 \mid \hat{y}) = \frac{P^G(\hat{y} \mid y=1)P(y=1)}{P^G(\hat{y} \mid y=1)P(y=1) + P^G(\hat{y} \mid y=0)(1-P(y=1))} \quad (2)$$

where $\hat{y} = f(\vec{x})$. Note that:

- $P^G(y = 1|\hat{y})$ is the probability that a patient actually belongs to the $y=1$ class, given that the clinical risk model, f, assigns a score of $\hat{y}$ to a patient with prognostic features, $\vec{x}$.
- Similarly, $P^G(\hat{y}|y = 1)$ is the probability that the risk score, f, assigns a score of $\hat{y}$ to patients in the positive class, and
- $P^G(\hat{y}|y = 0)$ is the probability that f assigns a score of $\hat{y}$ to patients in the negative class.

Since f is completely determined by $\vec{x}$, the associated univariate probability densities are:

$$\begin{aligned} \rho^G(\hat{y} \mid y=1) &= \int_{\vec{x}'} N(\vec{x}' \mid \mu_{y=1}, \Sigma_{y=1})\,\delta(\hat{y} - f(\vec{x}'))\,d\vec{x}' \\ \rho^G(\hat{y} \mid y=0) &= \int_{\vec{x}'} N(\vec{x}' \mid \mu_{y=0}, \Sigma_{y=0})\,\delta(\hat{y} - f(\vec{x}'))\,d\vec{x}' \end{aligned} \quad (3)$$

where δ is the Dirac delta function and ρ^G is the univariate pdf. The needed probabilities, $P^G(\hat{y}|y = 1)$ and $P^G(\hat{y}|y = 0)$ are calculated by numerically integrating the univariate probability densities.

With these conventions, our unreliability metric, $U(\vec{x})$, is obtained by comparing $\hat{y}$ and $P^G(\hat{y}|y = 1)$:

$$U(\vec{x}) \equiv \left| P^G(y=1 \mid \hat{y}) - \hat{y} \right| \quad (4)$$

It is straightforward to show that $U(\vec{x}) \neq 0$ implies $P^G(y = 1|\vec{x}) \neq f(\vec{x})$ (see Section II). Furthermore, it follows that $0 \leq U(\vec{x}) \leq 1$, where the higher the value of $U(\vec{x})$ the more unreliable the model prediction.

To arrive at a simplified expression for $U(\vec{x})$ we first define:

$$g(\gamma,\hat{y}) \equiv \frac{P(\hat{y}\mid y=1)\gamma}{P(\hat{y}\mid y=1)\gamma + P(\hat{y}\mid y=1)(1-\gamma)} = \frac{1}{1+\beta_{\vec{x}}^{-1}\left(\frac{1-\gamma}{\gamma}\right)} \text{ where } \beta_{\vec{x}} \equiv \frac{P(\hat{y}|y=1)}{P(\hat{y}|y=0)} \tag{5}$$

We refer to $\beta_{\vec{x}}$ as the relative likelihood.

Then we have:

$$\begin{aligned} g(\gamma,\hat{y}) = \hat{y} &\Rightarrow \frac{1}{1+\beta_{\vec{x}}^{-1}\left(\frac{1-\gamma}{\gamma}\right)} = \hat{y} \\ &\Rightarrow 1+\beta_{\vec{x}}^{-1}\left(\tfrac{1-\gamma}{\gamma}\right) = \tfrac{1}{\hat{y}} \\ &\Rightarrow 1+\beta_{\vec{x}}^{-1}\left(\tfrac{1}{\gamma}-1\right) = \tfrac{1}{\hat{y}} \\ &\Rightarrow \tfrac{1}{\gamma} = \beta_{\vec{x}}\left(\tfrac{1}{\hat{y}}-1\right)+1 \\ &\Rightarrow \gamma = \gamma_{\vec{x}} \equiv \left(\beta_{\vec{x}}\left(\tfrac{1}{\hat{y}}-1\right)+1\right)^{-1} = \left(\tfrac{\beta_{\vec{x}}}{\hat{y}} - \beta_{\vec{x}} + 1\right)^{-1} \end{aligned} \tag{6}$$

It follows that:

$$\begin{aligned} U(\vec{x}) &= \left|P^{G}(y=1\mid\hat{y}) - \hat{y}\right| \\ &= \left|g(P(y=1),\hat{y}) - g(\gamma_{\vec{x}},\hat{y})\right| \\ &= \left|\frac{1}{1+\beta_{\vec{x}}^{-1}\left(\frac{1-P(y=1)}{P(y=1)}\right)} - \frac{1}{1+\beta_{\vec{x}}^{-1}\left(\frac{1-\gamma_{\vec{x}}}{\gamma_{\vec{x}}}\right)}\right| \\ &= \left|\frac{1+\beta_{\vec{x}}^{-1}\left(\frac{1-\gamma_{\vec{x}}}{\gamma_{\vec{x}}}\right) - 1 - \beta_{\vec{x}}^{-1}\left(\frac{1-P(y=1)}{P(y=1)}\right)}{\left(1+\beta_{\vec{x}}^{-1}\left(\frac{1-P(y=1)}{P(y=1)}\right)\right)\left(1+\beta_{\vec{x}}^{-1}\left(\frac{1-\gamma_{\vec{x}}}{\gamma_{\vec{x}}}\right)\right)}\right| \\ &= \frac{\left|\beta_{\vec{x}}^{-1}\left(\frac{1-\gamma_{\vec{x}}}{\gamma_{\vec{x}}}\right) - \beta_{\vec{x}}^{-1}\left(\frac{1-P(y=1)}{P(y=1)}\right)\right|}{\left(1+\beta_{\vec{x}}^{-1}\left(\frac{1-P(y=1)}{P(y=1)}\right)\right)\left(1+\beta_{\vec{x}}^{-1}\left(\frac{1-\gamma_{\vec{x}}}{\gamma_{\vec{x}}}\right)\right)} \\ &= \frac{\left|\beta_{\vec{x}}^{-1}\left(\frac{1}{\gamma_{\vec{x}}}-1\right) - \beta_{\vec{x}}^{-1}\left(\frac{1}{P(y=1)}-1\right)\right|}{\left(1+\beta_{\vec{x}}^{-1}\left(\frac{1-P(y=1)}{P(y=1)}\right)\right)\left(1+\beta_{\vec{x}}^{-1}\left(\frac{1-\gamma_{\vec{x}}}{\gamma_{\vec{x}}}\right)\right)} \\ &= \frac{\left|\beta_{\vec{x}}^{-1}\left(\frac{1}{\gamma_{\vec{x}}} - \frac{1}{P(y=1)}\right)\right|}{\left(1+\beta_{\vec{x}}^{-1}\left(\frac{1-P(y=1)}{P(y=1)}\right)\right)\left(1+\beta_{\vec{x}}^{-1}\left(\frac{1-\gamma_{\vec{x}}}{\gamma_{\vec{x}}}\right)\right)} \end{aligned} \tag{7}$$

Substituting for $\gamma_{\bar{x}}$ we get:

$$
\begin{aligned}
U(\bar{x}) &= \frac{\left|\beta_{\bar{x}}^{-1}\left(\frac{\beta_{\bar{x}}}{\hat{y}} - \beta_{\bar{x}} + 1 - \frac{1}{P(y=1)}\right)\right|}{\left(1+\beta_{\bar{x}}^{-1}\left(\frac{1-P(y=1)}{P(y=1)}\right)\right)\left(1+\beta_{\bar{x}}^{-1}\left(\frac{\beta_{\bar{x}}}{\hat{y}} - \beta_{\bar{x}} + 1 - 1\right)\right)} \\
&= \frac{\left|\left(\frac{1}{\hat{y}} - 1 + \beta_{\bar{x}}^{-1} - \frac{\beta_{\bar{x}}^{-1}}{P(y=1)}\right)\right|}{\left(1+\beta_{\bar{x}}^{-1}\left(\frac{1-P(y=1)}{P(y=1)}\right)\right)\left(1+\beta_{\bar{x}}^{-1}\left(\frac{\beta_{\bar{x}}}{\hat{y}} - \beta_{\bar{x}} + 1 - 1\right)\right)} \\
&= \frac{\left|\frac{1}{\hat{y}} - 1 + \beta_{\bar{x}}^{-1} - \frac{\beta_{\bar{x}}^{-1}}{P(y=1)}\right|}{\left(1+\beta_{\bar{x}}^{-1}\left(\frac{1-P(y=1)}{P(y=1)}\right)\right)\frac{1}{\hat{y}}} \\
&= \hat{y}\frac{\left|\frac{1}{\hat{y}} - 1 + \beta_{\bar{x}}^{-1} - \frac{\beta_{\bar{x}}^{-1}}{P(y=1)}\right|}{\left(1+\beta_{\bar{x}}^{-1}\left(\frac{1-P(y=1)}{P(y=1)}\right)\right)} \\
&= \hat{y}\frac{\left|\frac{1}{\hat{y}} - 1 + \beta_{\bar{x}}^{-1}\left(1 - \frac{1}{P(y=1)}\right)\right|}{\left(1+\beta_{\bar{x}}^{-1}\left(\frac{1-P(y=1)}{P(y=1)}\right)\right)}
\end{aligned}
\tag{8}
$$

Hence the expression we use for our calculations is:

$$
U(\bar{x}) = \hat{y}\frac{\left|\left(\frac{1-\hat{y}}{\hat{y}}\right) - \beta_{\bar{x}}^{-1}\left(\frac{1-P(y=1)}{P(y=1)}\right)\right|}{\left(1+\beta_{\bar{x}}^{-1}\left(\frac{1-P(y=1)}{P(y=1)}\right)\right)}
\tag{9}
$$

II. **Theorem**: $U(x) \neq 0 \Rightarrow P^G\left(y=1|\vec{x}\right) \neq f(\vec{x})$

Taking the contrapositive, it is equivalent to show that if $P^G\left(y=1|\vec{x}\right) = f(\vec{x})$ then $U(x)=0$

First we find a simplified expression for $P^G(y = 1|\hat{y})$, where $\hat{y} = f(\vec{x})$.

$$P^G(y=1|\hat{y}) = \frac{P^G\left(y=1,\hat{y}\right)}{P^G(\hat{y})} = \frac{\int_{\vec{x}} P^G(y=1|\vec{x},\hat{y})P^G(\vec{x},\hat{y})d\vec{x}}{\int_{\vec{x}} P^G(\vec{x},\hat{y})d\vec{x}}$$

$$= \frac{\int_{f(\vec{x})=\hat{y}} P^G(y=1|\vec{x},\hat{y})P^G(\vec{x},\hat{y})d\vec{x}}{\int_{f(\vec{x})=\hat{y}} P^G(\vec{x},\hat{y})d\vec{x}} \quad \text{because } P(\vec{x},\hat{y})=0 \text{ when } \hat{y} \neq f(\vec{x})$$

$$= \frac{\int_{f(\vec{x})=\hat{y}} P^G(y=1|\vec{x})P^G(\vec{x},\hat{y})d\vec{x}}{\int_{f(\vec{x})=\hat{y}} P^G(\vec{x},\hat{y})d\vec{x}} \quad \text{because } P(y=1|\vec{x},\hat{y}) = P(y=1|\vec{x},f(\vec{x})) = P(y=1|\vec{x})$$

Suppose $P^G(y=1|\vec{x}) = \hat{y}$. Then by definition, $\forall\left(f(\vec{x})=\hat{y}\right): \hat{y} = P(y=1|\vec{x})$. Then,

$$\frac{\int_{f(\vec{x})=\hat{y}} P^G(y=1|\vec{x})P^G(\vec{x},\hat{y})d\vec{x}}{\int_{f(\vec{x})=\hat{y}} P^G(\vec{x},\hat{y})d\vec{x}} = \frac{\int_{f(\vec{x})=\hat{y}} \hat{y}P^G(\vec{x},\hat{y})d\vec{x}}{\int_{f(\vec{x})=\hat{y}} P^G(\vec{x},\hat{y})d\vec{x}}$$

$$= \hat{y}\frac{\int_{f(\vec{x})=\hat{y}} P^G(\vec{x},\hat{y})d\vec{x}}{\int_{f(\vec{x})=\hat{y}} P^G(\vec{x},\hat{y})d\vec{x}}$$

$$= \hat{y}$$

Hence $P^G\left(y=1|\hat{y}\right) = \hat{y}$ and $U(\vec{x})=0$

Q.E.D

III. Normalized Brier Scores for a binary outcome

We denote the outcome class label to be, $y_i \in \{0,1\}$, where $y_i = 1$ if and only if patient i belongs to the positive class. The prevalence of the outcome in the population is given by $\overline{y} = \frac{N^+}{N}$ where N is the number of patients in the population and N^+ is the number of patients who are in the positive class. The error of the null model is:

$$
\begin{aligned}
B_{null} &= \frac{1}{N}\sum_{i=1}^{N}\left(y_i - \overline{y}\right)^2 \\
&= \frac{1}{N}\sum_{i=1}^{N}\left(y_i^2 - 2y_i\overline{y} + \overline{y}^2\right) \\
&= \frac{1}{N}\left(\sum_{i=1}^{N} y_i^2 - 2\overline{y}\sum_{i=1}^{N} y_i + N\overline{y}^2\right) \\
&= \frac{1}{N}\left(N^+ - 2\overline{y}N^+ + N\overline{y}^2\right) \\
&= \frac{N^+}{N} - \frac{2\overline{y}N^+}{N} + \overline{y}^2 \\
&= \overline{y} - 2\overline{y}^2 + \overline{y}^2 \\
&= \overline{y} - \overline{y}^2
\end{aligned}
$$

The normalized Brier Score is given by B / B_{null} where $B = \frac{1}{N}\sum_{i=1}^{N}\left(y_i - f(x_i)\right)^2$. As the B lies between 0 and 1, the normalized Brier score, B / B_{null}, lies between 0 and $\left(\overline{y} - \overline{y}^2\right)^{-1}$.

SUPPLEMENTARY TABLE

Table 1: Characteristics of patients with predictions in the upper 1% of unreliability scores

	**Most Unreliable Predictions for GRACE Risk Model (Upper 1%)**	**Most Unreliable Predictions for the Stroke Risk Model (Upper 1%)**
**Demographics**		
Age in years (IDR[1])	84.1 (68.4-93.9)	68.3 (50.3-87.7)
Female	43.3	41.0%
Height in cm (IDR)	165 (150-182)	168 (154-180)
Admission weight in kg (IDR)	69.4 (45-97)	73.8 (50.0-99.5)
**Medical History Including Cardiac Risk Factors (%)**		
Angina	36.3	37.3
Congestive heart failure	28.7	6.6
Coronary Artery Bypass Graft	9.0	6.6
Diabetes	25.3	25.3
Hyperlipidemia	29.1	36.7
Hypertension	63.2	65.7
Myocardial Infarction	33.2	30.7
Percutaneous Coronary Intervention	8.6	18.7
Peripheral artery disease	12.0	12.0
Renal insufficiency	18.5	4.2
Smoking	34.1	53.6
TIA/Stroke	10.8	9.6

[1]IDR=Interdecile Range

SUPPLEMENTARY FIGURES

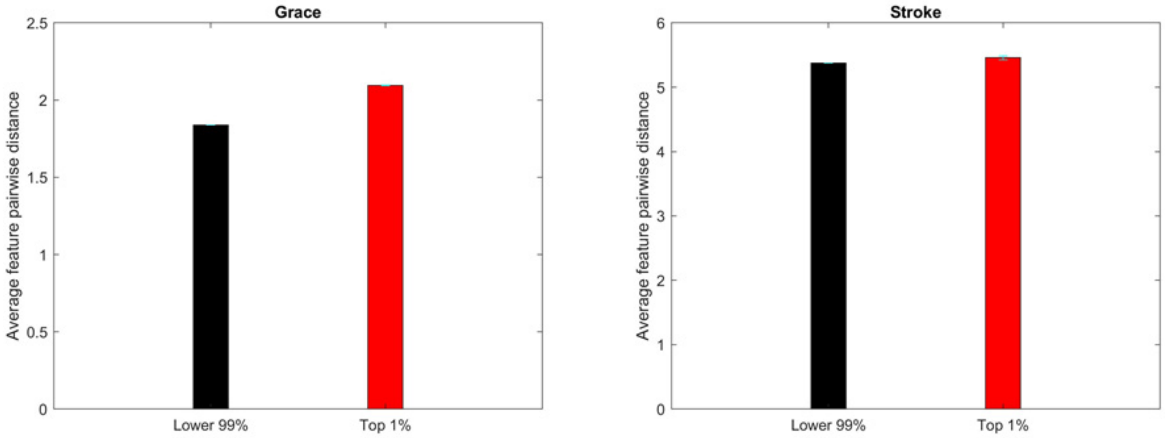

Figure 1: Average pairwise distance between features of patients who fall within the top 1% of uncertainty and those that fall within the remainder of the dataset. Patients who have predictions that fall within the highest 1% of uncertainty form a set that has features that are at least as heterogeneous as the set containing patients who are not in this subgroup.

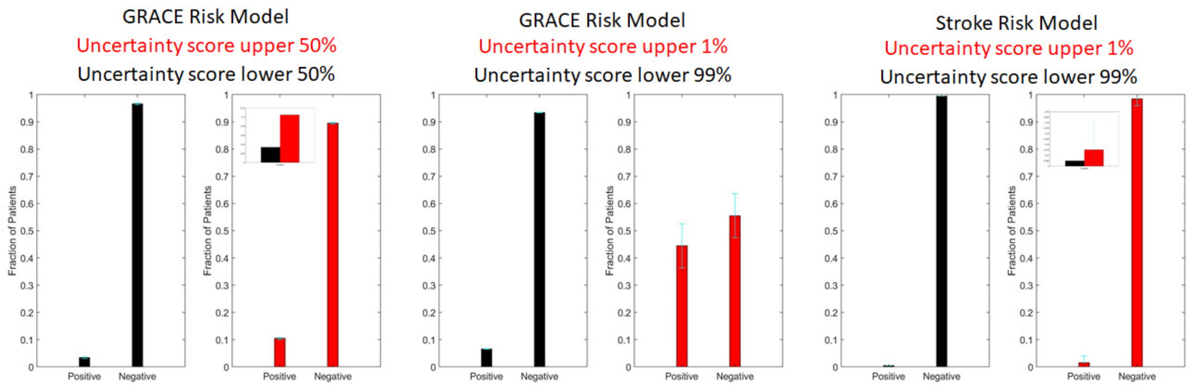

Figure 2: Relative prevalence of positive outcomes in patients in different cohorts, defined by their uncertainty score. Patients in cohorts with high uncertainty are more heterogeneous with respect to the outcome than patients who have lower uncertainty scores. Insets juxtapose the prevalence of positive and negative patients within relevant subgroups.

Supplementary References

1 Gupta, S. S. Probability Integrals of Multivariate Normal and Multivariate t^1. *The Annals of Mathematical Statistics* **34**, 792-828 (1963).
